# EFFECTS OF DIFFERENT PERIODS OF GASTRIC ISCHAEMIA IN THE VIABILITY OF THE TISSUE OF BODY, FUNDUS AND ANTRUM REGION OF RABBIT STOMACH

**DOI:** 10.1590/S0102-67202015nahead00001

**Published:** 2015-08-04

**Authors:** Maria Angélica B. MAGALHÃES, Alfredo J. A. BARBOSA, Juliano A. FIGUEIREDO, Luiz R. ALBERTI, Andy PETROIANU

**Affiliations:** 1 Laboratory of Digestive Pathology and Neuroendocrine, Department of Pathology, School of Medicine, Federal University of Minas Gerais, Belo Horizonte, MG; 2 Department of Surgery, Faculty of Medicine, Federal University of Minas Gerais,Belo Horizonte, MG; 3 Department of Veterinary Medicine at the Catholic University of Paraná, Curitiba PR, Brazil

**Keywords:** Gastric ischaemia, Gastric devascularization, Rabbit, Stomach, Gastric infarction

## Abstract

**BACKGROUND:**

Despite the rich vascular arcade of the stomach, gastric ischemia represents an important medical challenge and can be the consequence of obstructive or non-obstructive vascular processes of pathological or iatrogenic origin.

**AIM:**

To assess the effects of acute gastric ischaemia on the different regions of the stomach.

**METHOD:**

Fifteen New Zeland rabbits were divided into three groups: group 1, animals were observed during 3 h; group 2, during 6 h; group 3, during 12 h. Rabbit stomachs were subjected to devascularization of the greater and lesser curvatures. After predetermined time, the stomachs were removed for macro and microscopic studies.

**RESULTS:**

Haemorrhagic necrosis was more marked in the gastric fundus and body. In contrast, the antropylorus remained preserved in 80% of the animals. Necrosis of the gastric body and fundus mucosa were observed in all animals after 6 h and 12 h of ischaemia.

**CONCLUSION:**

Acute gastric ischaemia in rabbits produces haemorrhagic necrosis of the gastric fundus and body even in a short period of time. Beside this, the antropyloric region was significantly more resistant to ischaemia.

## Abstract (pt)

**RACIONAL:**

Isquemia gástrica representa importante desafio médico e geralmente é decorrente de processos vasculares obstrutivos ou não-obstrutivos. Apesar da rica arcada vascular do estômago, lesões de isquemia gástrica têm sido observadas. Quando a isquemia progride, pode culminar em necrose do órgão.

**OBJETIVO:**

Avaliar os efeitos dos diferentes tempos de isquemia gástrica sobre os tecidos da parede do estômago de coelhos mediante desvascularização total de ambas as curvaturas gástricas.

**MÉTODO:**

Foram utilizados 15 coelhos machos da raça Nova Zelândia. Os animais foram distribuídos nos seguintes grupos: grupo 1, observados por 3 h; grupo 2, por 6 h; grupo 3, por 12 h. A técnica cirúrgica foi a mesma em todos os animais e consistiu na ligadura e secção de todas as veias e artérias da parede gástrica. Após o período pré-determinado de observação, o estômago foi removido por completo para estudo macro e microscópico.

**RESULTADOS:**

As alterações macroscópicas e histológicas tornaram-se mais intensas com o aumento do tempo de isquemia e foram mais evidentes nas regiões do fundo e do corpo. Por outro lado, o antro manteve-se preservado na maioria dos animais operados. Necrose de mucosa do corpo e do fundo foi observada em todos os animais estudados, e foi acompanhada por hemorragia em 60% dos coelhos dos grupos 2 e 3.

**CONCLUSÃO:**

O modelo experimental de isquemia gástrica foi eficaz para produzir necrose hemorrágica de fundo e corpo do estômago de coelhos mesmo em curto tempo. Por outro lado, a região do antro pilórico desses animais mostrou-se significativamente mais resistente à isquemia do que as demais regiões.

## INTRODUCTION

The response of the gastric wall to ischemia was still not investigated in the proportion of its importance. Ischemia may be due to obstruction of the arterial or venous blood flow to the stomach mainly due to thromboembolism, phlebitis and gastric volvus^1^^,^ 2. Non-obstructive factors such as sepsis, congestive heart failure, side effects of digitalis and alpha-adrenergic agents may provoke a reduction of cardiac output, tissue hypoperfusion and gastric ischemia^2^^,^ 3. Furthermore, the stomach is an organ subjected to a large number of surgical procedures which are suitable to provoke gastric ischemia. Among these surgeries, some are worth to be stressed, such as surgical correction of portal hypertension, reversion of gastric volvus, subtotal gastrectomies to treat gastric neoplasias, esophagogastroplasties to reconstruct the digestive tube after esophagectomies and gastroplasties of obese patients^4^^-^ 7 **.** The most severe complication of gastric ischemia is necrosis of the stomach. 

Despite the large number of surgical procedures that interfere with the vascularization of the stomach and their complications, few studies dealing with acute gastric ischemia have been published. 

The objective of this study was to verify the effects of acute gastric ischemia on the different parts of the stomach. 

## METHOD

This experiment was carried out according to the ethical norms for animal experimentation and was approved by the Ethics Committee for Animal Experimentation of the Federal University of Minas Gerais, Belo Horizonte, Brazil under the Protocol nr. 145/2009.

Fifteen white New Zealand male rabbits weighing 2500 to 3000 g were anaesthetized with an injection into the gluteal region of 2% xylazine at the dose of 10 mg/kg in combination with 10% ketamine at the dose of 60 mg/kg^8^^,^ 9. The same surgical technique was used for all animals. The entire gastric vasculature, including veins and arteries, was ligated and sectioned along the greater and lesser curvature of the stomach, with the organ fixed in place only through the esophagus and duodenum, and laparorrhaphy was then performed. 

The animals were divided into three groups (n=5) according to time of observation after surgery: 3 h (group 1), 6 (group 2) and 12 h (group 3). After the predetermined periods of observation, the animals were killed with an overdose of the anesthetic followed by an intravenous injection of 10% potassium chloride. The stomachs were removed en bloc for macro- and microscopic studies.

The removed stomachs were opened along the greater curvature and washed with running water. Changes compatible with hemorrhagic necrosis of the fundus, body and antrum were recorded and fragments of these areas and of pre-established areas of each gastric region were collected, fixed in 4% formaldehyde, processed for paraffin embedding, and stained with hematoxylin and eosin (H&E) for histological study.

## RESULTS

**Figure 1:**
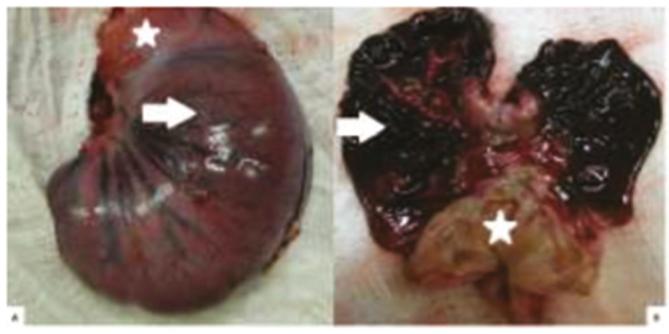
Macroscopic aspect of the closed and opened stomach of the same rabbit after 6 h of gastric ischemia: A) outer surface of the stomach with signs of hemorrhagic necrosis of the body (arrow), while the antropylorus (*) is preserved; B) diffuse hemorrhagic necrosis in the body and fundus (arrow), while the antrum (*) is preserved

**Figure 2:**
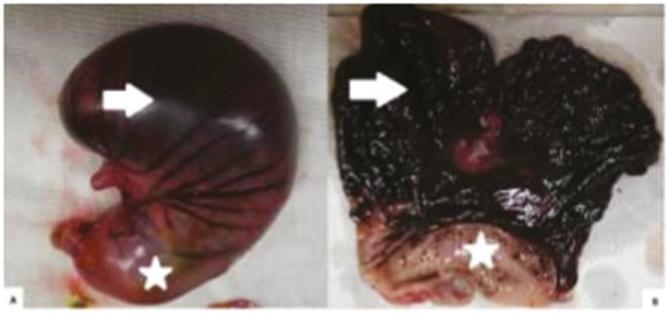
Macroscopic aspect of the closed and opened stomach of the same rabbit after 12 h of gastric ischemia: A) outer surface of the stomach with signs of hemorrhagic necrosis of the body and fundus (arrow) while the antropylorus (*) is preserved; B) diffuse and marked hemorrhagic necrosis of the gastric mucosa in the body and fundus (arrow), while the antrum (*) is preserved

Macroscopic examination of the stomach of all the animals in the three groups showed localized or diffuse changes indicative of hemorrhagic necrosis (Figures 1 and 2). The gastric fundus and body were most affected in all rabbits. In most animals the antral region was preserved, presenting only mild congestive changes of the mucosa in one animal each in groups 2 and 3. All animals of groups 1, 2 and 3 presented macroscopic lesions indicative of hemorrhagic necrosis of varying extension in the body and fundus, of greater intensity in groups 2 and 3 (6 and 12 h) compared to group 1 (3 h) (Table 1).

**Table 1:** Macroscopic aspect of localized or diffuse hemorrhagic necrosis in the rabbits gastric antrum, body and fundus submitted to ischemia for 3 (group 1), 6 (group 2) and 12 (group 3) hours

GROUPS (n = 5)	ANTRUM		BODY		FUNDUS	
	Localizedn (%)	Diffusen (%)	Localizedn (%)	Diffusen (%)	Localizedn (%)	Diffusen (%)
1	-	-	2 (40%)	3 (60%)	2 (40%)	3 (60%)
2	-	-	1 (20%)	4 (80%)	-	5 (100%)
3	-	-	-	100%	-	5 (100%)

Microscopic changes in stomach tissues became more conspicuous with increasing time of gastric ischemia. Edema and vessel congestion were observed in the mucosa, submucosa and muscle layers of the three gastric regions in all animals. Areas of mucosal necrosis of the body and fundus occurred in all animals after three hours of gastric ischemia and only one animal in this group presented necrosis of the muscle layer in the region of the gastric fundus (Table 2). 

**Table 2:** Microscopic aspects of the rabbit stomach showing hemorrhagic necrosis in the mucosal and muscle layers of the antrum, body and fundus after 3 (group 3), 6 (group 3) and 12 (group 3) hours of gastric ischemia

GROUPS (n=5)	ANTRUM		BODY		FUNDUS	
	Mucosan (%)	Musclen (%)	Mucosan (%)	Musclen (%)	Mucosan (%)	Musclen (%)
1	-	-	5 (100%)	-	5 (100%)	1 (20%)
2	1 (20%)	-	5 (100%)	2 (40%)	5 (100%)	2 (40%)
3	1 (20%)	-	5 (100%)	3 (60%)	5 (100%)	4 (80%)

Necrosis of the mucosa of the antropyloric region was observed in only one (20%) rabbit each in groups 2 and 3, and the muscle layer of this region was preserved in all animals of the three groups; hemorrhagic necrosis of the mucosa of the gastric body and fundus was observed in all animals of groups 2 (6 h) and 3 (12 h). Two animals (40%) developed necrosis of the muscle layer in the gastric body and fundus after 6 h of ischemia (group 2). After 12 h of ischemia (group 3), necrosis of the muscle layer was observed in the gastric body of 3 (60%) animals and in the fundus of 4 (80%) animals (Table 2). 

## DISCUSSION

Gastric ischemia is frequently followed by a precarious prognosis. Despite the rich vascular arcade of the stomach, complications of ischemic origin affecting this organ have been frequent in humans, due in part to the increasing number of abdominal operations that interfere with the vascularization of this organ. The symptoms of gastric ischemia are usually nonspecific, ranging from local pain to an intense acute abdomen^10^. Even without a precise diagnosis, surgical treatment must be rapid, otherwise mortality can be superior to 80%^11^ **.** Resection of necrotic segment is the appropriate treatment and total gastrectomy may be sometimes necessary^10^.

The effects of gastric devascularization on the vitality of the stomach have been evaluated in few experimental studies. In dogs, complete devascularization of the stomach wall may result in gangrene of the organ and cause death of the animals^12^. In humans, partial stomach devascularization is a frequent procedure. Ligation of the right and left gastric veins and those of the right and posterior upper wall of the stomach are the treatments adopted to reduce the hypertension of gastric varices^13^. Acute hemorrhagic lesions of the gastroduodenal mucosa refractory to clinical treatment represent other indications for local devascularization of the stomach^14^^-^ 16. Venous obstruction alone can have the same ischemic effects as arterial obstruction on the gastric wall. Venous thrombosis, phlebitis and coagulopathies are predisposing factors of ischemia^17^. 

In this study, devascularization was performed in the entire wall of the stomach, including all veins and arteries. The results obtained showed that the model of gastric ischemia was effective in all animals, as confirmed by macro- and microscopic examination, which revealed varied degrees of necrosis of the gastric wall. Gastric ischemia, even when induced for a relatively short period of time, may cause severe and irreversible injuries to one or more different tissues of the stomach. 

As observed, the gastric fundus and body were the regions more sensitive to ischemia, wheras the antrum was preserved in practically all animals. Since all the vessels of the greater and lesser curvatures were sectioned, the most likely explanation for the preservation of the antropyloric region is based on two possibilities. The first refers to the rich vascular anastomosis present in the gastroduodenal interface. These anastomosis derive from small branches of the gastroduodenal artery, which derives from the hepatic artery and are largely responsible for the formation of the vascular plexuses present in the submucosa of the more distal regions of the stomach. Submucosal microvessels originating from the duodenum probably provide supplementary blood irrigation to the gastric antrum, leading to a greater resistance to ischemia. This vascular distribution is well known to occur in humans and is closely similar to that of rabbits^18^^-^ 20. In addition, the anatomical variations of each individual may contribute to the maintenance of blood irrigation in specific regions of the stomach^21^. Thus, it is possible that the results obtained with this experimental model may be worth to be applied to humans. Secondly, the preservation of the gastric antrum vitality in the animals studied here may be explained by the relatively short time of induced ischemia (3 to 12 h). Since this region of the stomach appears to benefit from a larger number of vascular anastomoses, it may resist to general gastric ischemia for a longer period of time. This possibility is supported by the macro- and microscopic findings, which revealed lesions of greater intensity in the upper gastric regions even when the time of ischemia was increased to 12 h.

## CONCLUSION

Gastric ischemia produces hemorrhagic necrosis of the gastric fundus and body wall in rabbits; however, the antropylorus is preserved during the early postoperative period, indicating this part of the stomach is more resistant to ischemia.
